# Closed-Loop Phase-Dependent Vibration Stimulation Improves Motor Imagery-Based Brain-Computer Interface Performance

**DOI:** 10.3389/fnins.2021.638638

**Published:** 2021-01-25

**Authors:** Wenbin Zhang, Aiguo Song, Hong Zeng, Baoguo Xu, Minmin Miao

**Affiliations:** ^1^The State Key Laboratory of Bioelectronics, School of Instrument Science and Engineering, Southeast University, Nanjing, China; ^2^School of Information Engineering, Huzhou University, Huzhou, China

**Keywords:** brain-computer interface, closed-loop system, phase-dependent, motor imagery, vibration stimulation

## Abstract

The motor imagery (MI) paradigm has been wildly used in brain-computer interface (BCI), but the difficulties in performing imagery tasks limit its application. Mechanical vibration stimulus has been increasingly used to enhance the MI performance, but its improvement consistence is still under debate. To develop more effective vibration stimulus methods for consistently enhancing MI, this study proposes an EEG phase-dependent closed-loop mechanical vibration stimulation method. The subject’s index finger of the non-dominant hand was given 4 different vibration stimulation conditions (i.e., continuous open-loop vibration stimulus, two different phase-dependent closed-loop vibration stimuli and no stimulus) when performing two tasks of imagining movement and rest of the index finger from his/her dominant hand. We compared MI performance and brain oscillatory patterns under different conditions to verify the effectiveness of this method. The subjects performed 80 trials of each type in a random order, and the average phase-lock value of closed-loop stimulus conditions was 0.71. It was found that the closed-loop vibration stimulus applied in the falling phase helped the subjects to produce stronger event-related desynchronization (ERD) and sustain longer. Moreover, the classification accuracy was improved by about 9% compared with MI without any vibration stimulation (*p* = 0.012, paired *t*-test). This method helps to modulate the mu rhythm and make subjects more concentrated on the imagery and without negative enhancement during rest tasks, ultimately improves MI-based BCI performance. Participants reported that the tactile fatigue under closed-loop stimulation conditions was significantly less than continuous stimulation. This novel method is an improvement to the traditional vibration stimulation enhancement research and helps to make stimulation more precise and efficient.

## Introduction

Brain-computer interface (BCI) systems provide users with a non-muscular channel to send messages or instructions to external devices using brain activities (Wolpaw et al., 2002). Moreover, BCI based on electroencephalography (EEG) signal has attracted wide attention due to its non-invasiveness, convenience, and high time resolution. The widely used EEG-based BCI paradigms include event-related P300 potentials, steady-state visually evoked potentials (SSVEPs), motor imagery (MI), etc. (Sharma et al., 2006; Polich, 2007; Vialatte et al., 2010; Zhang et al., 2017, 2019; Jiang et al., 2019, 2020). Among these paradigms, MI is an active BCI that allows users to adjust their alpha/beta rhythm to generate features by imagining limb movements (Pfurtscheller and Neuper, 2001). Extensive research has proved that the neurophysiological basis of motor imagery is that when subjects imagine the left- or right-hand movement, they will generate event-related desynchronization (ERD) in the specific frequency bands (commonly mu and beta) of the contralateral sensorimotor areas and event-related synchronization (ERS) on the ipsilateral side (Pfurtscheller and Lopes da Silva, 1999; Pfurtscheller et al., 2006; Tecchio et al., 2006).

However, researchers indicate that there are about 15–30% of the subjects who cannot reach proficiency in using BCI, which is called “BCI-illiteracy” (Guger et al., 2003; Minkyu et al., 2013). And the MI-based BCI has some other problems, such as the imbalance between the dominant and non-dominant hands (Maruff et al., 1999), inconsistent individual performance (Ahn and Jun, 2015), etc. Therefore, more and more researchers are devoted to improving MI-based BCI performance. The main research directions include improving MI decoding accuracy through optimizing feature extraction and classification algorithms (Lotte et al., 2018; Chen et al., 2020; Hang et al., 2020); proposing new hybrid BCI by introducing additional neural signals (such as EMG (electromyogram) (Vaughan et al., 1998; Leeb et al., 2011), fNIRS (functional near-infrared spectroscopy) (Wang et al., 2019), and gaze (Punsawad et al., 2010; Zeng et al., 2017), multiple brain modes (such as P300 and SSVEP) or multiple sensory stimuli (visual, auditory, tactile, etc.) (Pfurtscheller et al., 2010).

Among them, the study of combining sensory input with MI has achieved many exciting results, Allison et al. designed a hybrid BCI that combines the SSVEP and ERD features, which improves the decoding rate by about 6% compared to the traditional MI-based BCI (Allison et al., 2010). Cincotti et al. (2007) compared the effects of visual and vibrotactile feedback on subjects. Compared with visual feedback, the advantage of tactile feedback for MI is that it does not occupy the visual channel and retains the advantage of the MI paradigm. Meanwhile, it allows subjects to adjust their brain activities by themselves, especially for users with impaired or missing vision. Muller et al. demonstrated the feasibility of the tactile-based BCI paradigm and achieved 70% accuracy by selectively sensation of the vibration stimulation applied to the left and right index fingers (Muller-Putz et al., 2006). The fusion of tactile stimuli and MI has been proved in previous studies to improve MI-based BCI performance, specifically in reducing imagination time, increasing decoding accuracy, etc. (Chatterjee et al., 2007; Yao et al., 2014, 2015; Yi et al., 2017). Furthermore, physiological studies have found that tactile stimuli applied to the hand on the imaginary side can enhance the activation of the contralateral cortex (Nobuaki et al., 2011; Mizuguchi et al., 2012). Shu et al. (2017) found that applying unilateral tactile stimulation to non-dominant/paralyzed hands could increase MI-induced ERD lateralization potential.

Nevertheless, most researches on hybrid BCI that combine tactile and MI uses continuous, open-loop stimulation, often applied at a fixed time with a pre-set frequency and intensity (Pfurtscheller et al., 2010; Yi et al., 2017). It cannot be adjusted according to the real-time state of the subjects. In contrast, the closed-loop system can apply stimulation with the changes in the subject’s status. In such a system, the phase has essential effects on the real-time state of the brain. Many studies have shown that stimulation for the brain will produce different effects according to the applied phase. For example, the application of transcranial magnetic stimulation (TMS) pulses at the peak or trough of the mu rhythm of the motor cortex have opposite plastic effects (Zrenner et al., 2017). Fehér et al. (2017) demonstrated a phase-dependent modulatory mechanism of tACS at a cortical network level. Similarly, Holt et al., 2019 used intracranial electrical stimulation based on the β-rhythm of EEG in patients with Parkinson’s disease to adjust the amplitude of β-oscillation (Holt et al., 2019). The frequency and phase-specific effects of transcranial alternating current stimulation have also been shown in many experiments involving motor activity (Guerra et al., 2016; Nakazono et al., 2016), cognitive (Polanía et al., 2012), and auditory systems (Riecke et al., 2015; Polanía et al., 2018).

Among the phase-dependent researches, the alpha oscillations is particularly concerned. Lindsley first proposed that the brain state might be reflected by the phase of alpha oscillations in a phasic form (Lindsley, 1952). A phase-dependent stimulation system may potentially make a stronger effect of the stimulation on the subject or make the direction of the effect more precise, but it still lacks relevant research. Ai and Ro’s (2014) research proves that 8–12 Hz neural oscillations in the somatosensory areas can affect tactile perception, and that pulsed inhibition by these oscillations shapes the state of brain activity necessary for conscious perception. Therefore, whether the close-loop vibration stimulation based on the alpha phase will affect the performance of MI-based BCI or not is worth studying.

In order to investigate the effect of vibration stimuli applied on a specific phase interval on MI BCI, we proposed a phase-based closed-loop vibration stimulation method and designed a comparative experiment to compare the difference between this method and the traditional continuous open-loop vibration stimulation methods independent of EEG phase on motor imagery. These experiments include pure motor imagery without stimulation (PMI), open-loop continuous vibration stimulation (CVS), vibration stimulation in the rising phase (RPS), and vibration stimulation in the falling phase (FPS). The oscillation modes and time-frequency characteristics of EEG under different stimulation conditions were compared, and feature extraction was performed using the FBCSP algorithm to verify whether this method based on closed-loop vibration stimulation can improve the performance of the MI-based BCI.

## Materials and Methods

### Subjects

Ten healthy right-handed subjects (8 males, 2 females, aged range 21–28 years) participated in the experiment. The hand dominance of subjects was determined by the Edinburgh Handedness Inventory. None of the subjects have prior experience with BCIs. Before the experiment, they were informed about the experimental procedure and signed the informed consent before participating in the experiment. They were not informed of the purpose of applying vibration stimulation to avoid the subject’s psychological state bias when performing the imagination task. The study was approved by the Ethics Committee of Southeast University. All subjects received experimental compensation.

### Tactile Stimulation

A total of three stimulation methods were designed: continuous vibration stimulation, vibration stimulation applied to the rising phase or the falling phase. Vibration stimulation provided by C2 voice coil tactor from Engineering Acoustics, Inc. (Winter Park, FL), applied to the fingertip of the non-dominant hand of the subject. The tactor was placed in a customized rubber soft base to reduce the noise generated by the desktop’s resonance when stimulating. The Pacinian corpuscles and Meissner corpuscles in the mechanical receptor of human fingertips are sensitive to frequencies above 100 and 20–50 Hz, respectively (Breitwieser et al., 2012). To drive the tactor, the PC soundcard produced a 23 Hz sine wave modulated with a 200 Hz sine carrier wave and amplified with an audio amplifier. The vibration intensity was controlled by changing the volume until the subject feel the vibration obviously without affecting the imagination.

The phase-dependent vibration stimulation is applied to the rising ([−π/6, π/3]) or falling ([5 × π/6, 4 × π/3]) phase of the alpha oscillations in the C4 channel. Each trigger vibrates at 200 Hz frequency for 20 ms. This study used MATLAB to collect data and calculate the current phase once every 40 ms. Whether to trigger the stimulus or not is determined by the predicted current phase. The interval between each stimulation is more than 100 ms. Using the “tic-tic-toc” pattern (Severens et al., 2010) (the amplitude of the third vibration after every two beats is increased by 50%) to help the subjects maintain their attention to the vibration.

### Experimental Procedure

In this study, subjects executed the tasks of motor imagery under different stimulus conditions, as shown in Figure 2. The experiment was divided into 4 sessions, namely, motor imagery without stimulus (PMI), motor imagery with continuous vibration stimulus (CVS), and motor imagery with phase-dependent vibration stimulus in rising (RPS) or falling intervals (FPS). Each session contains two runs, each run contains 40 trials, in which the non-dominant hand finger-tapping task and the relaxation task were executed in random order of 20 each. Each run lasted about 7 min, the subjects had about 10 min break between every two runs. Before the start of the formal experiment, the subjects were required to complete one run of the motor execution task and one run of the MI task for training, to help the subjects familiarize themselves with the experimental procedure and adequately understand the motor imagery task. In order to avoid learning effects, each subject completed four sessions in random order and completed all experiments on the same day.

**FIGURE 1:**
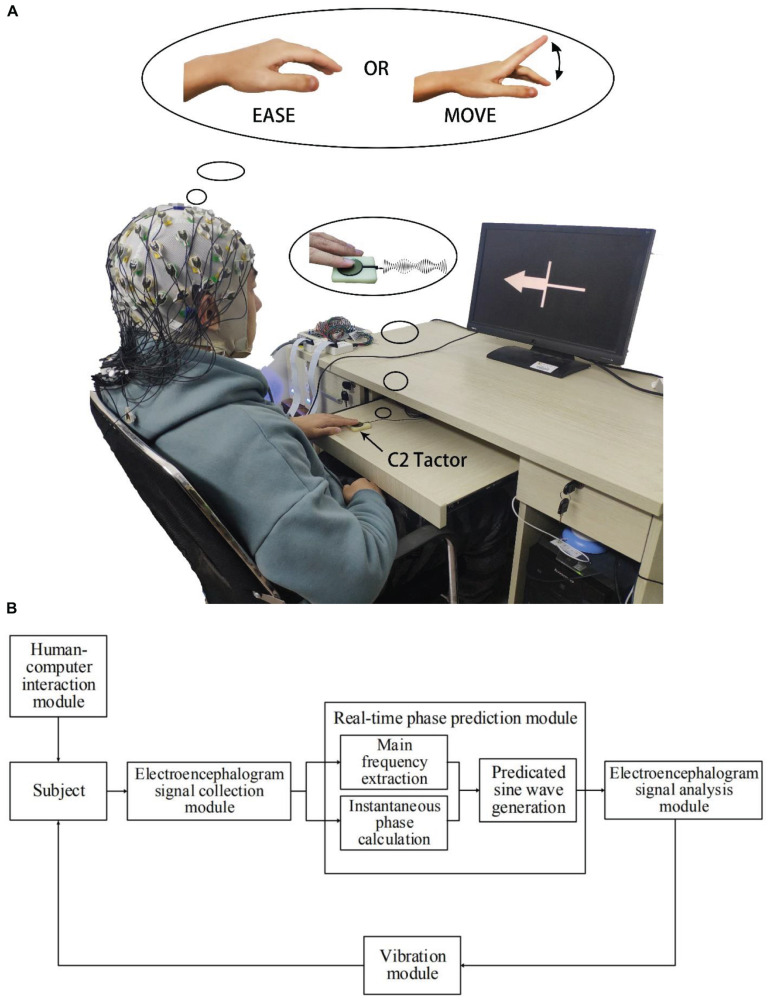
**(A)** Experimental setup: The subjects were seated in a comfortable chair. Their non-dominant hand’s index finger was placed on the C2 tactor, and they performed imagination tasks according to the instructions displayed on the screen. The tactor provides corresponding vibration stimulation according to the experimental settings. **(B)** Flowchart of the closed-loop stimulation system.

**FIGURE 2:**
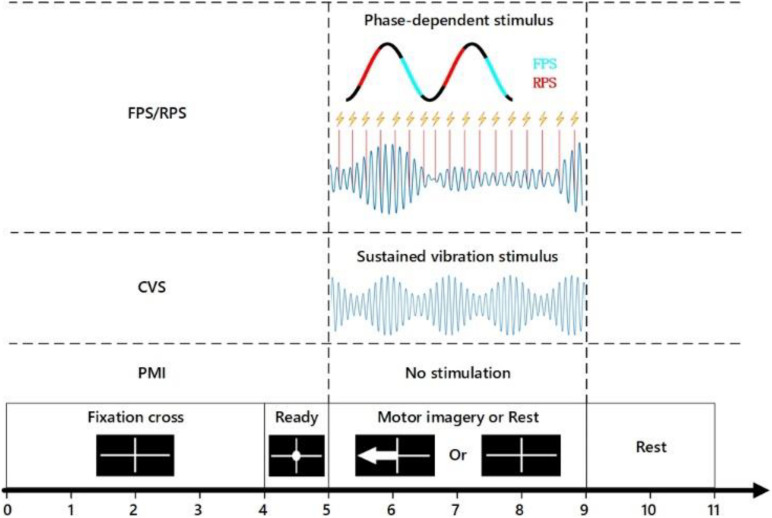
Experimental procedure of the four different sessions, all sessions use the same time structure. The only difference is the way the mechanical vibration stimulus applied to the subject’s fingertip within 5–9 s: under FPS/RPS conditions, a 20 ms vibration stimulus was applied each time according to the phase of EEG alpha wave. Under FPS/RPS conditions, the constant 23 Hz vibration stimulus with 200 Hz as the carrier frequency was applied to the fingertip of the subjects. No stimulation was applied under PMI conditions.

During the experiment, all subjects were sitting in a comfortable chair about 1 meter away from the monitor. Put the non-dominant hand wrist on the platform and put the index finger on the tactor, both hands were relaxed as shown in Figure 1. To avoid placebo effects, the subjects were asked to place their fingers on the tactor through all sessions. Figure 2 illustrates the experimental paradigm of four different sessions (PMI/CVS/RPS/FPS). The time structure of all sessions is the same as the MI task, but the applied vibration stimuli were different. At the beginning of each trial, there was a white cross displayed in the center of the screen, the subjects can relax, and a white circle appears in the middle of the cross at the 4th second, prompting the subjects to pay attention to the imagination task that was about to start for 1 s. At the 5th second, the subjects perform the motion imagery task according to the arrow or cross displayed on the screen. If the arrow pointing to the left appeared, the subject performs the imagination of the non-dominant hand index finger tapping. If the white cross appeared, the subject performs the rest task, which continued for 4 s. Then the screen was black, the subjects can relax until the white cross appears at the 11th second to prompt the start of the next trial. To minimize artifacts, subjects were asked to avoid physical movements and blinking during the task. The time structure of all sessions was the same, only the tactile stimulus applied during the 5–9 s is different.

### EEG Recording and Phase-Tracking Approach

A SynAmps2 amplifier (Neuroscan Compumedics, United States) and a 64-channel active electrode cap (BrainAmp, ActiCap, BrainProducts, Munich, Germany) were used to acquire the EEG signal. We recorded 27 channels (Fp1, Fp2, F1, Fz, F2, FC1-6, FCz, C1-6, Cz, CP1-6, CPz, P1, P2, Pz) based on International 10/20 system (see Figure 2). The reference electrode was placed on the left mastoid and the electrode located on the forehead served as the ground. All the impedance of electrodes was kept below 10 kΩ during the recordings. An analog bandwidth filter with 0.1–100 Hz and a notch filter with 50 Hz were applied to the EEG signals. The EEG sampling rate was set to 1,000 Hz.

In order to deliver tactile stimulation output at a specified phase interval of a given frequency, we have adopted a previously proposed method based on Fast Fourier Transform (FFT) to track the phase of the EEG signal in real-time (Farrokh et al., 2017). The EEG signals were transmitted from the amplifier to MATLAB (MathWorks, United States) in real-time through the TCP / IP protocol for real-time phase tracking. Starting from the 5th second of each trial, extract the latest 300 ms data of the C4 channel every 40 ms and perform the following steps to predict the phase of the current time point: First, a 10th order elliptical infinite impulse response (IIR) filter is used to perform 8–12 Hz bandpass filtering the data; second, the FFT of this data segment is calculated; third, calculate the frequency and phase of the dominant component of the signal from the FFT; finally, using a simple sine function to forecast the signal by using the calculated phase and frequency.

### Algorithms and Analysis Methods

In this study, the data were analyzed off-line using customized MATLAB programs and the MATLAB-based EEGLAB toolbox (Delorme and Makeig, 2004). To remove the artifacts caused by eye movements, the automatic artifact removal (AAR) toolbox with the SOBI algorithm was used (Gómez-Herrero, 2007). Afterward, common average reference (CAR) was adopted to re-reference the data. Event-related spectral perturbation (ERSP) was used to evaluate the mean spectral power changes in time-frequency and spatial domains. In this work, the ERSP of n trials was calculated by Eq. (1):

(1) $$E\,R\,S\,P\,\left(f,t\right)=\frac{1}{n}\,\sum_{k=1}^{n}\left(F_{k}\,{\left(f,t\right)}^{2}\right)$$

Where *F*_*k*_(*f*,*t*) indicates the spectral estimation of kth trial at frequency *f* and time *t*, n is the number of trials. We use EEGLAB to compute the ERSP (dB), the short-time Fourier transform (STFT) was applied with a Hanning-tapered window which was length 200 ms. To normalize the baseline, each spectral estimation subtracted the mean power changes in [3 4] s (the 1 s epoch before attention cue appear). The key electrodes C3 and C4 were selected to display the time courses from 0 to 9 s between 1 and 30 Hz. The ERD/ERS values were calculated in alpha (8–13 Hz) and beta (14–30 Hz) respectively.

To compare the MI-BCI classification performance under different feedback conditions, all 27 channels data were utilized for pattern classification, and the data of 0.5–4 s after imagine task onset were extracted for feature extraction and pattern classification. The specific effect of tactile stimulation is not clear, and we cannot make the *priori* choice of the relevant frequency band(s), so the Filter Bank Common Spatial Pattern (FBCSP) algorithm (Ang et al., 2008; Keng et al., 2012) was utilized to extract the features from the narrowband EEG signals for classification. FBCSP divides each epoch of EEG data into sub-bands with different frequency bands and then implement the CSP algorithm on the filtered signals at each sub-band to calculate the corresponding features. In this study, the sub-bands are chosen from the range 8–32 Hz with bandwidth of 4 Hz (8–12, 12–16, 16–20, 20–24, 24–28, 28–32 Hz). After that, we use Fisher’s linear discriminative analysis (LDA) for the 2-class classification.

For statistical analysis, a 10 × 10-fold cross-validation strategy was utilized to evaluate the classification performance. Every group includes two runs, 80 trials and corresponding categories were randomly divided into 10 sets, each consist of 8 trials. Among the 10 sets, each 9 parts (72 trials) were for training the LDA classifier and the remaining one part (8 trials) was for testing. Repeating 10 times to get 100 results, and finally get the average classification accuracy. To evaluate the performance of the phase-tracking algorithm, we calculated the Hilbert transformation offline and compared the phase using the Phase Locking Value (PLV).

## Results

**FIGURE 3:**
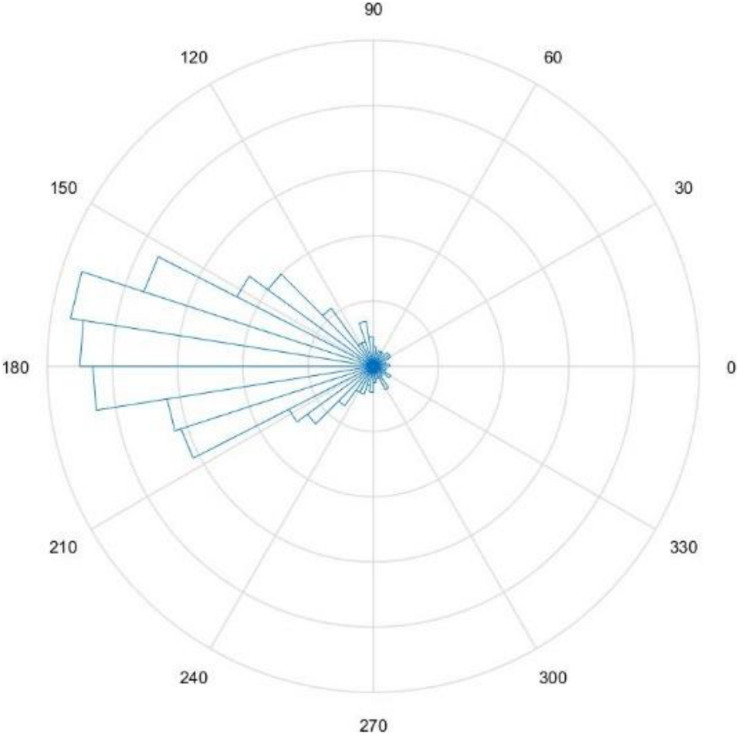
Rose plot under FPS conditions, shows the actual phase of the vibration stimulus.

The main purpose of this study is to investigate whether phase-based closed-loop vibration tactile stimulation can improve the performance of the MI paradigm BCI. The MI-BCI applied with closed-loop vibration stimulation based on the rising or falling phase of the EEG signal in real-time was compared with the MI-BCI without stimulation or with open-loop stimulation, respectively. The offline analysis evaluated the phase-tracking algorithm based on FFT was used to obtain PLV = 0.71, and the trials with more errors in the applied stimulation position were removed. Figure 3 shows a rose plot of the stimulus phase of one subject’s FPS run.

### Event-Related Spectral Perturbation (ERSP)

**FIGURE 4:**
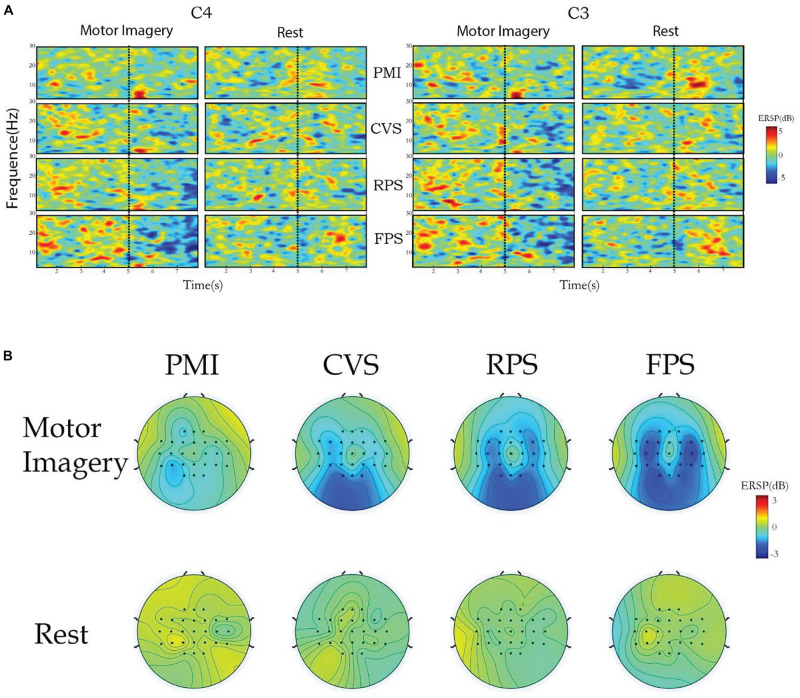
The cortical activations in time-frequency **(A)** and spatial **(B)** domains for subject S2.

Figure 4 shows the ERSP distributions across time-frequency (A) and spatial domains (B) from one representative subject (S2). The C3 and C4 channels were selected to cover the left and right sensorimotor cortices in time-frequency analysis. No obvious desynchronization was observed for the imagery tasks in the alpha and beta bands of the PMI task. In contrast, CVS, RPS, and FPS all enhance the activation of the sensorimotor cortex to varying degrees. However, the frequency band of continuous stimulus activation is narrow, mainly concentrated in the alpha band, and the level of activation is not as deep as the closed-loop stimulus. And FPS shows some time accumulation effects, the activation is broader and stronger as the imagine time progresses. Regarding closed-loop stimulation, it can be observed that the FPS task has a better effect on cortical activation than RPS, especially in the C4 channel. But for the rest task, it showed no obvious cortical activation in C3 and C4, and even more obvious ERS phenomenon was observed on both sides of the FPS task. CVS and RPS tasks can observe some discrete activations in the alpha band, but the range and intensity are smaller than imagery. Moreover, through the topographic maps, it is clearly shown that, compared with the weak ERD of the PMI task, the three tasks that introduce vibration stimulation significantly enhance the activation of the contralateral and ipsilateral sensorimotor cortex. The enhancement of the closed-loop stimulation’s activation range and intensity are more obvious than those of the CVS task. As observed, CVS did not produce strong enough activation on the contralateral side, while FPS produced the strongest activation on both C3 and C4 channels. Meanwhile, no matter whether vibration stimulation was applied or not, no obvious cortical activation was observed during the rest. Only PMI and FPS tasks observed slight ERS on the ipsilateral sensorimotor cortex. It can be seen that tactile stimulation does not directly activate the sensorimotor cortex, but rather promotes and enhances the cortical activation induced by motor imagery.

**FIGURE 5:**
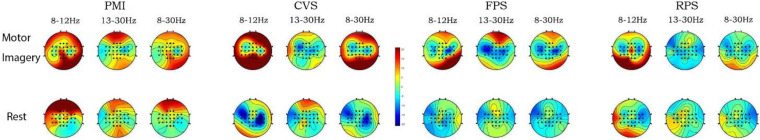
Grand-averaged distributions of ERD patterns of all subjects for each class. The EEG power was averaged from three representative frequency bands (8–12; 13–30; 8–30 Hz) over 4 after the imagery task begins.

The averaged topographical distributions of ERD topographic maps across all subjects are shown in Figure 5. The first row shows the activation patterns of motor imagery under four different conditions, and the second row is the rest. There are three frequency ranges under each condition (8–12, 13–30, and 8–30 Hz correspond to the alpha, beta, and alpha-beta rhythms, respectively), which makes the effects of different tactile stimuli on MI-based BCI at different frequencies more intuitively. During the left-hand motor imagery, the contralateral sensorimotor cortex shows a certain degree of activation in all conditions. All three kinds of vibration stimulation significantly enhanced the desynchronization of this area. Clear cortical desynchronization can be observed at both alpha and beta rhythm. Simultaneously, the ipsilateral sensorimotor cortex activation is also enhanced to a certain extent, especially in the beta band. Importantly, a significant desynchronization of the bilateral sensorimotor cortex is observed in the alpha band of the CVS task in the rest class, which is significantly stronger than the activation during motor imagery. This may indicate that some subjects were directly activated by continuous vibration stimulation, or involuntarily imagine the movement of their limbs during the stimulation. Besides, some activation of the contralateral sensorimotor cortex can be seen in the alpha band under the FPS and RPS tasks, but the intensity is much lower than that of CVS and the activation of motor imagery under the same conditions.

### Classification Performance

**FIGURE 6:**
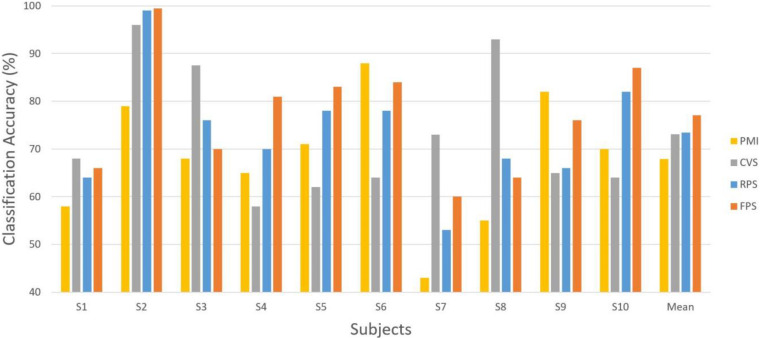
Classification accuracies and mean accuracies in four conditions over 10 subjects.

Off-line classification accuracies across all the subjects with their mean accuracies are shown in Figure 6, and the best averaged classification accuracy of cross-validation is chosen as the performance. It is observed that the classification performance in FPS tasks is significantly better than that of PMI tasks (*p* = 0.012, paired *t*-test), but there is no significant difference in performance between PMI and CVS tasks, nor between FPS and RPS tasks. Compared with PMI tasks, the average performance of FPS tasks increased by about 9%, and compared with CVS and RPS tasks, it increased by about 4%. In FPS tasks, the number of BIC-illiterate subjects (accuracy < 70%) decreased from 6 to 3 out of 10 subjects. Nevertheless, under other task conditions, the number has not decreased significantly. In terms of average classification performance, tactile vibration stimulation generally enhances MI-based BCI, but individual performance is different. Among them, the performance of two subjects on the PMI task is higher than that of the other vibration stimulation tasks, while the classification performance of three subjects in the CVS tasks is significantly higher than the closed-loop vibration stimulation tasks and PMI tasks, which will be discussed further in the discussion section.

## Discussion

### Effects of Tactile Stimulation on MI-BCI

In this study, we explored the impact of vibration stimulation on MI-BCI. Tactile feedback has been increasingly used in BCI research due to its good human-computer interaction and the characteristics of reducing dependence on visual channels. There is mainly mechanical vibration stimulation, kinesthetic stimulation, electrical stimulation, and so on in terms of stimulation types. Among them, somatokinetic stimulation is more natural than other methods, which is in line with people’s understanding and expectations of actual actions. However, it often requires the assistance of larger devices or is placed in a specific environment. Electrical stimulation is more powerful than vibration stimulation and can transmit the stimulation to nerves more effectively, but many subjects have fear and resistance to it. In contrast, the vibration stimulation selected in this study has the advantages of easy wearing, low cost, and high acceptance by subjects, and is more suitable for MI-based BCI. Many studies have demonstrated that the introduction of vibrotactile stimulation can increase the excitability of the motion-related cortex (Mizuguchi et al., 2013), and have proved that the vibration stimulation can enhance the performance of the motor imagination paradigm, but further research is needed on the details of the application method.

First of all, the stimulus application method: Some studies use short-term vibration stimuli to prompt the beginning and ending of the imagery task or to inform the users of the classification results (Chatterjee et al., 2007; McCreadie et al., 2014; Jeunet et al., 2015). This kind of stimulus feedback method is often used to replace visual feedback and has little effect on classification performance. Therefore, many recent studies have focused on applying continuous tactile stimulation during imagination, and the effect of enhancing the performance of pure MI-BCI is different. Yao et al. (2013) designed a hybrid BCI that combines motor imagery and selective sensation using vibration stimuli applied by both hands simultaneously based on selective perception. Compared with the PMI task, the performance of hybrid BCI was not significantly improved. However, most of the subjects whose classification accuracy had not improved were subjects who had performed well in PMI. The subjects whose MI classification accuracy was below 60% had a visible improvement after the introduction of vibration stimulation. Similarly, Ahn et al. (2014) also studied a tactile hybrid BCI based on selective sensation. It also showed no enhancement of MI performance under bilateral vibration stimulation conditions, but the classification accuracy of MI that was executed after 3 seconds of selective sensation to vibration stimulation was about 10% higher than that of pure MI. Combined with the fact that in this study, under CVS conditions, the classification accuracy of some subjects was significantly decreased, which may be due to the difficulty of the subjects to focus on the two tasks of selective sensation and motor imagery at the same time. In this case, the vibration stimulation interfered with the participants’ imagination of the action. However, under the same conditions of continuous stimulation of the hands, Yi et al. (2017) used continuous electrical stimulation and combined selective sensation with MI, but improved the overall performance of an MI-based BCI, achieving 14% improvement in total relative to the MI task alone. The reason is that continuous electrical stimulation induces additional steady-state somatosensory evoked potentials (SSSEP). The SSSEP does not directly enhance ERD, but motor imagery will affect SSSEP, resulting in more selective sensation results and making the hybrid BCI perform better. It can be seen that the effects of applying continuous vibration stimulation while performing MI may be both pros and cons. On the one hand, it has been shown that tactile stimulation can directly induce alpha/beta ERD (Gaetz and Cheyne, 2006). And the spatial attention to tactile stimulation can further regulate the activation of the cortex (Bauer et al., 2006; Dockstader et al., 2010). High-intensity tactile stimulation combined with selective sensation to induce SSSEP can further generate more features to improve classification performance. However, it is worth noting that most of the pure selective sensation paradigms using vibration stimulation only achieve a decoding rate of about 60% (Yao et al., 2013; Ahn et al., 2014; Shu et al., 2018), which shows that the ERD and other features produced by MI still play an important role in hybrid BCI. On the other hand, vibration stimulation may not induce SSSEP due to insufficient intensity or different individual adaptation frequencies. At the same time, it may distract the subjects and interfere with the subjects’ imagery tasks.

Compared with stimulating both hands at the same time and then combined with selective sensation, it seems more effective to apply tactile stimuli to the imaginary ipsilateral hand (Chatterjee et al., 2007). Shu et al. (2017) applied constant tactile stimulation to the non-dominant hand improved the imbalance of MI ability between the dominant hand and the non-dominant hand, and improved the classification accuracy of MI-BCI by about 11%. This study also applied vibration stimuli to the non-dominant hand, but unlike other studies, it distinguished between imagining non-dominant hand movement and rest. Physiological studies have proved that applying tactile stimuli on the side of imagery can enhance the activation of the contralateral cortex, but no obvious cortical changes are observed on the contralateral hand. Vibration stimulation has a higher intensity than somatosensory stimulation, such as holding a ball. In addition to providing subjects with a more vivid imagination environment and increasing the excitability of the motor cortex (de Moraes Silva et al., 2015), vibration stimulation itself will also activate the sensory cortex (Gaetz and Cheyne, 2006; Tu-Chan et al., 2017). Therefore, applying a fixed vibration stimulus to one hand will definitely affect the EEG mode of the rest state and affect the classification effect.

### Enhancement of Closed-Loop Vibration Stimulation on MI

**FIGURE 7:**
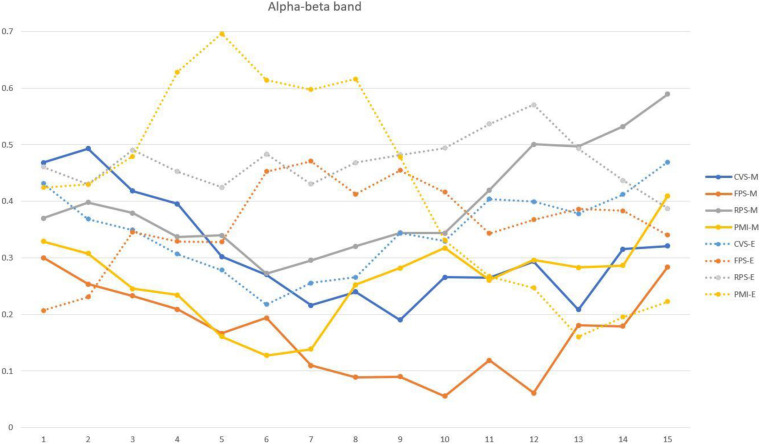
The average energy fluctuation graph of all subjects in the alpha-beta frequency band (8–30 Hz) within 4 s after the start of the imagery task. A window with a length of 1 s is calculated every 200 ms. The solid lines correspond to imagery tasks under different conditions, and the dashed lines correspond to rest tasks.

In this work, our research focuses on the impact of closed-loop vibration stimulation on MI-BCI. Compared with open-loop vibration stimulation, closed-loop stimulation shows better performance in classification accuracy and the magnitude of ERD. The physiological basis for phase-based closed-loop vibration stimulation to be effective for MI is that neural oscillations in the range of 8–12 Hz can affect tactile perception (Ai and Ro, 2014), and pulses of the sensorimotor cortex alpha rhythm can promote corticospinal excitability. Previous studies on non-invasive precise phase stimulation generally used electrical stimulation, TMS, and other stimulation methods. However, sensory stimuli such as vision and hearing are increasingly used in real-time stimulation, and the effect is pronounced (Dijk et al., 2008; Romei et al., 2010). This study used the algorithm proposed by Farrokh et al. (2017) to estimate the real-time phase of the EEG of the contralateral sensorimotor cortex (C4 channel) and distinguished the rising and falling intervals to apply vibration stimulation. Studies have shown that the perception of vibration stimuli at the alpha peak is inhibited in terms of the impact on stimulus perception, while the valley is more sensitive (Ai and Ro, 2014). Schaworonkow et al. (2018) found that the MEP amplitude is modulated by the mu phase in a wide range of stimulation intensity, and the stimulation applied to the negative peak has the best effect. The intensity of the stimulation will also affect the regulation of the mu rhythm cortex excitability. In addition, in terms of attention, even in a continuous spatial attention state, the perception results will change with phase changes (Helfrich et al., 2018). It can be seen from Figure 5 that the motor imagery task under FES conditions exhibits the strongest ERD mode, which means that FES conditions have the best effect on the enhancement of exercise-related cortical excitability, followed by RPS conditions. At the same time, it has been observed that imagining the non-dominant hand movement will cause the activation of the ipsilateral sensorimotor cortex, which is consistent with the results of previous studies (Porro et al., 2000). Furthermore, many studies have found that applying a stimulus before the trough helps to reduce the trough, while the stimulus applied at the rising phase cannot effectively increase the peak. Moreover, the descending phase interval stimulus has a cumulative effect, and continuous multiple stimuli can further reduce the amplitude (Holt et al., 2019). That may contribute to the continuation and enhancement of ERD to a certain extent. In order to verify this, we compared the average energy changes of all subjects during the motor imagery or rest task. As shown in Figure 7, in the MI task, the energy of the C4 channel under FPS conditions is the lowest, and it shows a downward trend as time progresses, which may be caused by the cumulative effect of stimulation mentioned before. In contrast, the energy decrease of pure MI in the first 2 s is not much different from FPS, but it does not continue to decrease, which may be due to the participant’s inability to keep focusing on their imagination. Energy reduction is also observed under CVS conditions and there is no rebound over time, but it is worth noting that the rest task under CVS conditions also shows energy decline, which is similar to the imagery task. This may be the main reason for the poor effect of CVS classification. The highest energy is observed in the rest task without stimulation, which indicates that vibration stimulation will induce the activation of the contralateral sensorimotor cortex no matter imagined or not. Among them, FPS makes the most prominent energy difference because an increase of task complexity or attention results in an increased magnitude of ERD (Boiten et al., 1992; Mashat et al., 2019).

The disadvantage of continuous open-loop vibration stimulation is that some subjects are not sensitive to mechanical vibration stimulation or only sensitive to specific vibration frequencies. In addition, the mechanical vibration stimulation of the same part for a long time will make some subjects feel tired and make the skin numb. To make the short-term vibration stimulation more easily perceivable, we applied 200 ms vibration stimulation every time and used the “tic-tic-toc” stimulation mode to enhance the participant’s attention to the stimulation (Gescheider et al., 2010; Choi et al., 2015). After completing the experiment, all the subjects reported that the closed-loop stimulation method could be clearly felt compared with CVS, and the degree of fatigue is lower. Besides, the frequency of stimulation may also be one of the reasons for the enhanced MI performance. The closed-loop stimulation is applied according to the phase of the alpha rhythm, so the actual stimulation frequency is also about 10 Hz. Previous research has shown that incoming tactile stimuli at an alpha frequency can enhance task-related alpha desynchronization (Long et al., 2016). Zhang et al. (2020) experimented with enhancing MI by electrically stimulating the ulnar nerve of the contralateral wrist at the alpha frequency (10 Hz), and improved the classification accuracy of left- and right-hand MI by about 15%. But in the actual application of motor imagery, the imagination task is often initiated by people subjectively rather than pre-set. Therefore, compared with online applications, it may be more suitable for assisting the training of MI. But this proves the effectiveness of alpha frequency tactile stimulation for MI-BCI enhancement from the side.

### Application Prospects and Future Works

This article tried to apply vibration stimuli in different phase intervals of real-time EEG signals, but the estimation algorithm of EEG real-time phase still has room for improvement in accuracy and computational efficiency. Physiological studies (Ai and Ro, 2014) have proved that the neural oscillation of the alpha band can affect sensory perception, so in addition to the phase information, we can try to monitor the activation of the sensory-motor cortex of the subject according to the activation state of the cerebral cortex and cortical energy. Further enhance the robustness and effectiveness of the closed-loop stimulation system and reduce vibration stimulation interference on the concentration of MI. Besides, because vibration stimulation may be particularly effective for BCI-illiteracy groups, while subjects with better MI performance are likely to have the opposite effect, the intensity or frequency of vibration stimulation should be adjusted adaptively according to the actual performance of the subjects (Bergmann et al., 2019).

In addition, some studies have shown that somatosensory stimulation can improve the motor function training effect of stroke patients (Sawaki et al., 2006; Bastos Conforto et al., 2010). The activation of the contralateral cortex during MI enhanced by vibration stimulation is also considered helpful to the neural reorganization of stroke patients, so closed-loop vibration stimulation can be further applied to assist stroke rehabilitation training. Limited by the small number of subjects, the effect of vibration stimulation applied in different intervals on cortical activation and energy changes is still unclear. In future works, more subjects should be recruited to further study the changes in cortical energy caused by vibration stimulation in different phase intervals, and to conduct long-term experiments to explore whether vibration stimulation has a long-term effective effect on MI-BCI.

## Conclusion

In this work, a closed-loop vibration stimulation method based on real-time phase prediction is proposed to improve MI-based BCI’s overall performance. The effects of continuous vibration stimulation and closed-loop vibration stimulation in two different phase intervals on the non-dominant hand imagination movement and rest classification performance are compared and analyzed. We found that the closed-loop vibration stimulation in the descending interval can deepen the contralateral ERD of MI to a greater extent than other conditions, thereby significantly improving the classification accuracy. Subjects generally report that closed-loop stimulation methods can better assist the execution of MI and are less prone to tactile fatigue. The method proposed in this paper is an improvement to the existing BCI enhancement methods. It may be expected to benefit people with complete somatosensory systems but impaired motor functions in the future.

## Data Availability Statement

The raw data supporting the conclusions of this article will be made available by the authors, without undue reservation.

## Ethics Statement

The studies involving human participants were reviewed and approved by the Ethics Committee of Southeast University. The patients/participants provided their written informed consent to participate in this study.

## Author Contributions

WZ, AS, and HZ designed the study. WZ set up the experiment platform. BX and WZ performed the experiment. WZ and MM analyzed the data and wrote the manuscript. AS, HZ, and BX were involved in critical revision of the manuscript. All the authors read and approved the final manuscript.

## Conflict of Interest

The authors declare that the research was conducted in the absence of any commercial or financial relationships that could be construed as a potential conflict of interest.

## References

[B1] Ahn M. Jun S. C. (2015). Performance variation in motor imagery brain–computer interface: a brief review. *J. Neurosci. Methods* 243 103–110. 10.1016/j.jneumeth.2015.01.033 25668430

[B2] Ahn S. Ahn M. Cho H. Jun S. C. (2014). Achieving a hybrid brain–computer interface with tactile selective attention and motor imagery. *J. Neural Eng.* 11:066004 10.1088/1741-2560/11/6/066004 25307730

[B3] Ai L. Ro T. (2014). The phase of prestimulus alpha oscillations affects tactile perception. *J. Neurophysiol.* 111 1300–1307. 10.1152/jn.00125.2013 24381028

[B4] Allison B. Z. Brunner C. Kaiser V. Müller-Putz G. R. Neuper C. Pfurtscheller G. (2010). Toward a hybrid brain-computer interface based on imagined movement and visual attention. *J. Neural Eng.* 7:26007. 10.1088/1741-2560/7/2/026007 20332550

[B5] Ang K. K. Chin Z. Y. Zhang H. Guan C. (2008). “Filter bank common spatial pattern (FBCSP) in brain-computer interface,” in *Proceedings of the IEEE International Joint Conference on Neural Networks*, (Hong Kong: IEEE). 10.1109/IEMBS.2009.5332817 19963715

[B6] Bastos Conforto A. Nocelo Ferreiro K. Tomasi C. dos Santos R. L. Loureiro Moreira V. Nagahashi Marie S. K. (2010). Effects of somatosensory stimulation on motor function after subacute stroke. *Neurorehabil. Neural Rep.* 24 263–272. 10.1177/1545968309349946 19884642 PMC2824782

[B7] Bauer M. Oostenveld R. Peeters M. Fries P. (2006). Tactile spatial attention enhances gamma-band activity in somatosensory cortex and reduces low-frequency activity in parieto-occipital areas. *Neuroscience* 26 490–501. 10.1523/jneurosci.5228-04.2006 16407546 PMC6674422

[B8] Bergmann T. O. Lieb A. Zrenner C. Ziemann U. (2019). Pulsed facilitation of corticospinal excitability by the sensorimotor mu-alpha rhythm. *J. Neurosci.* 39 10034–10043. 10.1523/jneurosci.1730-19.2019 31685655 PMC6978939

[B9] Boiten F. Sergeant J. Geuze R. (1992). Event-related desynchronization: the effects of energetic and computational demands. *Electroencephalogr. Clin. Neurophysiol.* 82 302–309. 10.1016/0013-4694(92)90110-4 1372551

[B10] Breitwieser C. Kaiser V. Neuper C. Müller-Putz G. R. (2012). Stability and distribution of steady-state somatosensory evoked potentials elicited by vibro-tactile stimulation. *Med. Biol. Eng. Comput.* 50 347–357. 10.1007/s11517-012-0877-9 22399162

[B11] Chatterjee A. Aggarwal V. Ramos A. Acharya S. Thakor N. V. (2007). A brain-computer interface with vibrotactile biofeedback for haptic information. *J. Neuroeng. Rehabil.* 4:40. 10.1186/1743-0003-4-40 PMC2104531 17941986

[B12] Chen Y. Hang W. Liang S. Liu X. Li G. Wang Q. (2020). A novel transfer support matrix machine for motor imagery-based brain computer interface. *Front. Neurosci.* 14:606949. 10.3389/fnins.2020.606949 33328874 PMC7719793

[B13] Choi I. Bond K. A. Krusienski D. J. Nam C. S. (2015). “Comparison of stimulation patterns to elicit steady-state somatosensory evoked potentials (SSSEPs): implications for hybrid and SSSEP-based BCIs,” in *Proceedings of the 2015 IEEE International Conference on Systems, Man, and Cybernetics (SMC)*, (Kowloon Tong: IEEE).

[B14] Cincotti F. Kauhanen L. Aloise F. Palomäki T. Capurusso N. Jylänki P. (2007). Vibrotactile feedback for brain-computer interface operation. *Comput. Intell. Neurosci.* 2007:48937. 10.1155/2007/48937 PMC2267023 18354734

[B15] de Moraes Silva J. Lima F. P. S. de Paula Júnior A. R. Teixeira S. Lima S. T. Bastos V. H. (2015). Assessing vibratory stimulation-induced cortical activity during a motor task-a randomized clinical study. *Neurosci. Lett.* 608 64–70. 10.1016/j.neulet.2015.09.032 26424076

[B16] Delorme A. Makeig S. (2004). EEGLAB: an open source toolbox for analysis of single-trial EEG dynamics including independent component analysis. *Neurosci. Methods* 134 9–21. 10.1016/j.jneumeth.2003.10.009 15102499

[B17] Dijk H. V. Schoffelen J. M. Oostenveld R. Jensen O. (2008). Prestimulus oscillatory activity in the alpha band predicts visual discrimination ability. *J. Neuroence Official J. Soc. Neurosci.* 28 1816–1823. 10.1523/jneurosci.1853-07.2008 18287498 PMC6671447

[B18] Dockstader C. Cheyne D. Tannock R. (2010). Cortical dynamics of selective attention to somatosensory events. *Neuroimage* 49 1777–1785. 10.1016/j.neuroimage.2009.09.035 19781649

[B19] Farrokh M. Katharine D. Peter G. Jonathan D. Zariffa J. (2017). A fast eeg forecasting algorithm for phase-locked transcranial electrical stimulation of the human brain. *Front. Neurosci.* 11:401. 10.3389/fnins.2017.00401 28775678 PMC5517498

[B20] Fehér K. D. Nakataki M. Morishima Y. (2017). Phase-dependent modulation of signal transmission in cortical networks through tACS-induced neural oscillations. *Front. Hum. Neurosci.* 11:471. 10.3389/fnhum.2017.00471 29021749 PMC5624081

[B21] Gaetz W. Cheyne D. (2006). Localization of sensorimotor cortical rhythms induced by tactile stimulation using spatially filtered MEG. *Neuroimage* 30 899–908. 10.1016/j.neuroimage.2005.10.009 16326116

[B22] Gescheider G. A. Wright J. H. Verrillo R. T. (2010). *Information-processing Channels in the Tactile Sensory System: A Psychophysical and Physiological Analysis*. Hove: Psychology Press.

[B23] Gómez-Herrero G. (2007). *Automatic Artifact Removal (AAR) Toolbox v1.3*. Tampere: Tampere University of Technology.

[B24] Guerra A. Pogosyan A. Nowak M. Tan H. Ferreri F. Di Lazzaro V. (2016). Phase dependency of the human primary motor cortex and cholinergic inhibition cancelation during beta tACS. *Cereb. Cortex* 26 3977–3990. 10.1093/cercor/bhw245 27522077 PMC5028010

[B25] Guger C. Edlinger G. Harkam W. Niedermayer I. Pfurtscheller G. (2003). How many people are able to operate an eeg-based brain-computer interface (bci)? *IEEE Trans. Neural Syst. Rehabil. Eng.* 11 145–147. 10.1109/tnsre.2003.814481 12899258

[B26] Hang W. Feng W. Liang S. Wang Q. Choi K. S. (2020). Deep stacked support matrix machine based representation learning for motor imagery eeg classification. *Comp. Methods Programs Biomed.* 193:105466. 10.1016/j.cmpb.2020.105466 32283388

[B27] Helfrich R. F. Fiebelkorn I. C. Szczepanski S. M. Lin J. J. Parvizi J. Knight R. T. (2018). Neural mechanisms of sustained attention are rhythmic. *Neuron* 99 854–865.e5. 30138591 10.1016/j.neuron.2018.07.032 PMC6286091

[B28] Holt A. B. Kormann E. Gulberti A. Pötter-Nerger M. McNamara C. G. Cagnan H. (2019). Phase-dependent suppression of beta oscillations in Parkinson’s disease patients. *J. Neurosci.* 39 1119–1134. 10.1523/jneurosci.1913-18.2018 30552179 PMC6363933

[B29] Jeunet C. Vi C. Spelmezan D. N’Kaoua B. Lotte F. Subramanian S. (2015). “Continuous tactile feedback for motor-imagery based brain-computer interaction in a multitasking context,” in *Paper presented at INTERACT 2015, Bamberg, Germany*, (Bamberg: Springer International Publishing).

[B30] Jiang Y. Zhang Y. Lin C. Wu D. Lin C. T. (2020). Eeg-based driver drowsiness estimation using an online multi-view and transfer tsk fuzzy system. *IEEE Trans. Intell. Transport. Syst.* 1–13.

[B31] Jiang Y. Zhao K. Xia K. Xue J. Zhou L. Ding Y. (2019). A novel distributed multitask fuzzy clustering algorithm for automatic mr brain image segmentation. *J. Med. Syst.* 43:118. 10.1007/s10916-019-1245-1 30911929

[B32] Keng A. K. Yang C. Z. Chuanchu W. Cuntai G. Haihong Z. (2012). Filter bank common spatial pattern algorithm on bci competition iv datasets 2a and 2b. *Front. Neurosci.* 6:39. 10.3389/fnins.2012.00039 22479236 PMC3314883

[B33] Leeb R. Sagha H. Chavarriaga R. Millán J. R. (2011). A hybrid brain-computer interface based on the fusion of electroencephalographic and electromyographic activities. *J. Neural Eng.* 8:025011 10.1088/1741-2560/8/2/025011 21436524

[B34] Lindsley D. B. (1952). Psychological phenomena and the electroencephalogram. *Electroencephalogr. Clin. Neurophysiol.* 4:443 10.1016/0013-4694(52)90075-8 12998592

[B35] Long J. Tazoe T. Soteropoulos D. S. Perez M. A. (2016). Interhemispheric connectivity during bimanual isometric force generation. *J. Neurophysiol.* 115:00876.2015. 10.1152/jn.00876.2015 PMC4808122 26538610

[B36] Lotte F. Bougrain L. Cichocki A. Clerc M. Congedo M. Rakotomamonjy A. (2018). A review of classification algorithms for EEG-based brain-computer interfaces: a 10 year update. *J. Neural Eng.* 15:031005 10.1088/1741-2552/aab2f2 29488902

[B37] Maruff P. Wilson P. H. Fazio J. D. Cerritelli B. Currie J. (1999). Asymmetries between dominant and non-dominant hands in real and imagined motor task performance. *Neuropsychologia* 37 379–384. 10.1016/s0028-3932(98)00064-5 10199649

[B38] Mashat M. E. M. Lin C. T. Zhang D. (2019). Effects of task complexity on motor imagery based brain-computer interface. *IEEE Trans. Neural Syst. Rehabil. Eng.* 27 2178–2185. 10.1109/tnsre.2019.2936987 31443036

[B39] McCreadie K. A. Coyle D. H. Prasad G. (2014). Is sensorimotor BCI performance influenced differently by mono, stereo, or 3-D auditory feedback? *IEEE Trans. Neur. Syst. Rehab.* 22 431–440. 10.1109/tnsre.2014.2312270 24691154

[B40] Minkyu A. Hohyun C. Sangtae A. Chan J. S. Dewen H. (2013). High theta and low alpha powers may be indicative of bci-illiteracy in motor imagery. *PLoS One* 8:e80886. 10.1371/journal.pone.0080886 24278339 PMC3838377

[B41] Mizuguchi N. Nakata H. Hayashi T. Sakamoto M. Muraoka T. Uchida Y. (2013). Brain activity during motor imagery of an action with an object: a functional magnetic resonance imaging study. *Neurosci. Res.* 76 150–155. 10.1016/j.neures.2013.03.012 23562793

[B42] Mizuguchi N. Sakamoto M. Muraoka T. Moriyama N. Kanosue K. (2012). Influence of somatosensory input on corticospinal excitability during motor imagery. *Neurosci. Lett.* 514 127–130. 10.1016/j.neulet.2012.02.073 22402190

[B43] Muller-Putz G. R. Scherer R. Neuper C. Pfurtscheller G. (2006). Steady-state somatosensory evoked potentials: suitable brain signals for brain-computer interfaces? *IEEE Trans. Neural Syst. Rehabil. Eng.* 14 30–37. 10.1109/tnsre.2005.863842 16562629

[B44] Nakazono H. Ogata K. Kuroda T. Tobimatsu S. (2016). Phase and frequency-dependent effects of transcranial alternating current stimulation on motor cortical excitability. *PLoS One* 11:e0162521. 10.1371/journal.pone.0162521 27607431 PMC5015848

[B45] Nobuaki M. Masanori S. Tetsuro M. Kento N. Shoichi K. Hiroki N. (2011). The modulation of corticospinal excitability during motor imagery of actions with objects. *PLoS One* 6:e26006. 10.1371/journal.pone.0026006 22022491 PMC3192791

[B46] Pfurtscheller G. Allison B. Z. Brunner C. Bauernfeind G. Birbaumer N. (2010). The hybrid bci. *Front. Neurosci.* 4:30. 10.3389/fnpro.2010.00003 20582271 PMC2891647

[B47] Pfurtscheller G. Brunner C. Schlögl A. Lopes da Silva F. H. (2006). Mu rhythm (de)synchronization and EEG single-trial classification of different motor imagery tasks. *Neuroimage* 31 153–159. 10.1016/j.neuroimage.2005.12.003 16443377

[B48] Pfurtscheller G. Lopes da Silva F. H. (1999). Event-related EEG/MEG synchronization and desynchronization: basic principles. *Clin. Neurophysiol.* 110 1842–1857. 10.1016/s1388-2457(99)00141-8 10576479

[B49] Pfurtscheller G. Neuper C. (2001). Motor imagery and direct brain-computer communication. *Proc. IEEE* 89 1123–1134. 10.1109/5.939829

[B50] Polanía R. Nitsche M. A. Korman C. Batsikadze G. Paulus W. (2012). The importance of timing in segregated theta phase-coupling for cognitive performance. *Curr. Biol.* 22 1314–1318. 10.1016/j.cub.2012.05.021 22683259

[B51] Polanía R. Nitsche M. A. Ruff C. C. (2018). Studying and modifying brain function with non-invasive brain stimulation. *Nat. Neurosci.* 21 174–187. 10.1038/s41593-017-0054-4 29311747

[B52] Polich J. (2007). Updating p300: an integrative theory of p3a and p3b. *Clin. Neurophysiol.* 118 2128–2148. 10.1016/j.clinph.2007.04.019 17573239 PMC2715154

[B53] Porro C. A. Cettolo V. Francescato M. P. Baraldi P. (2000). Ipsilateral involvement of primary motor cortex during motor imagery. *Eur. J. Neurosci.* 12 3059–3063. 10.1046/j.1460-9568.2000.00182.x 10971647

[B54] Punsawad Y. Wongsawat Y. Parnichkun M. (2010). “Hybrid EEG-EOG brain-computer interface system for practical machine control,” in *Proceedings of the International Conference of the IEEE Engineering in Medicine & Biology* (Buenos Aires: IEEE). 10.1109/IEMBS.2010.5626745 21096331

[B55] Riecke L. Formisano E. Herrmann C. S. Sack A. T. (2015). 4-Hz transcranial alternating current stimulation phase modulates hearing. *Brain Stimul.* 8 777–783. 10.1016/j.brs.2015.04.004 25981160

[B56] Romei V. Gross J. Thut G. (2010). On the role of prestimulus alpha rhythms over occipito-parietal areas in visual input regulation: correlation or causation? *J. Neurosci.* 30 8692–8697. 10.1523/jneurosci.0160-10.2010 20573914 PMC6634639

[B57] Sawaki L. Wu C. W.-H. Kaelin-Lang A. Cohen L. G. (2006). Effects of somatosensory stimulation on use-dependent plasticity in chronic stroke. *Stroke* 37 246–247. 10.1161/01.str.0000195130.16843.ac 16322491

[B58] Schaworonkow N. Triesch J. Ziemann U. Zrenner C. (2018). Eeg-triggered tms reveals stronger brain state-dependent modulation of motor evoked potentials at weaker stimulation intensities. *Brain Stimul.* 12 110–118. 10.1016/j.brs.2018.09.009 30268710

[B59] Severens M. Farquhar J. Desain P. Duysens J. Gielen C. (2010). Transient and steady-state responses to mechanical stimulation of different fingers reveal interactions based on lateral inhibition. *Clin. Neurophysiol.* 121 2090–2096. 10.1016/j.clinph.2010.05.016 21035742

[B60] Sharma N. Pomeroy V. M. Baron J. C. (2006). Motor imagery: a backdoor to the motor system after stroke? *Stroke* 37 1941–1952. 10.1161/01.str.0000226902.43357.fc 16741183

[B61] Shu X. Chen S. Meng J. Yao L. Sheng X. Jia J. (2018). Tactile stimulation improves sensorimotor rhythm-based bci performance in stroke patients. *IEEE Trans. Bio Med. Eng.* 66, 1987–1995. 10.1109/tbme.2018.2882075 30452349

[B62] Shu X. Yao L. Sheng X. Zhang D. Zhu X. (2017). Enhanced motor imagery-based BCI performance via tactile stimulation on unilateral hand. *Front. Hum. Neurosci.* 11:585. 10.3389/fnhum.2017.00585 29249952 PMC5717029

[B63] Tecchio F. Zappasodi F. Melgari J. M. Porcaro C. Rossini P. M. (2006). Sensory-motor interaction in primary hand cortical areas: a magnetoencephalography assessment. *Neuroscience* 141 533–542. 10.1016/j.neuroscience.2006.03.059 16713107

[B64] Tu-Chan A. P. Natraj N. Godlove J. Abrams G. Ganguly K. (2017). Effects of somatosensory electrical stimulation on motor function and cortical oscillations. *J. Neuroeng. Rehabil.* 14:113. 10.1186/s12984-017-0323-1 PMC5683582 29132379

[B65] Vaughan T. M. Miner L. A. Mcfarland D. J. Wolpaw J. R. (1998). Eeg-based communication: analysis of concurrent emg activity. *Electroencephalogr. Clin. Neurophysiol.* 107 428–433. 10.1016/s0013-4694(98)00107-2 9922089

[B66] Vialatte F.-B. Maurice M. Dauwels J. Cichocki A. (2010). Steady-state visually evoked potentials: focus on essential paradigms and future perspectives. *Prog. Neurobiol.* 90 418–438. 10.1016/j.pneurobio.2009.11.005 19963032

[B67] Wang Z. Zhou Y. Chen L. Gu B. Yi W. Liu W. (2019). Bci monitor enhances electroencephalographic and cerebral hemodynamic activations during motor training. *IEEE Trans. Neural Syst. Rehabil. Eng.* 27 780–787. 10.1109/tnsre.2019.2903685 30843846

[B68] Wolpaw J. R. Birbaumer N. Mcfarland D. J. (2002). Brain-computer interfaces for communication and control. *Clin. Neurophysiol.* 113 767–791. 12048038 10.1016/s1388-2457(02)00057-3

[B69] Yao L. Jianjun M. Sheng X. Zhang D. Zhu X. (2015). A novel calibration and task guidance framework for motor imagery bci via a tendon vibration induced sensation with kinesthesia illusion. *J. Neural Eng.* 12 113–123. 10.1088/1741-2560/12/1/016005 25461477

[B70] Yao L. Meng J. Zhang D. Sheng X. Zhu X. (2013). Selective sensation based brain-computer interface via mechanical vibrotactile stimulation. *PLoS One* 8:e64784. 10.1371/journal.pone.0064784 23762253 PMC3675213

[B71] Yao L. Meng J. Zhang D. Sheng X. Zhu X. (2014). Combining motor imagery with selective sensation toward a hybrid-modality bci. *Biomed. Eng. IEEE Trans.* 61 2304–2312. 10.1109/tbme.2013.2287245 24235291

[B72] Yi W. Qiu S. Wang K. Qi H. Zhao X. He F. (2017). Enhancing performance of a motor imagery based brain–computer interface by incorporating electrical stimulation-induced sssep. *J. Neural Eng.* 14:026002. 10.1088/1741-2552/aa5559 28004644

[B73] Zeng H. Wang Y. Wu C. Song A. Liu J. Ji P. (2017). Closed-loop hybridgaze brain-machine interface based robotic arm control with augmented reality feedback. *Front. Neurorobot.* 11:60. 10.3389/fnbot.2017.00060 29163123 PMC5671634

[B74] Zhang X. Guo Y. Gao B. Long J. (2020). Alpha frequency intervention by electrical stimulation to improve performance in mu-based bci. *IEEE Trans. Neural Syst. Rehabil. Eng.* 28, 1–1. 10.1155/2019/7030286 32305926

[B75] Zhang Y. Chung F. L. Wang S. (2019). A multiview and multiexemplar fuzzy clustering approach: theoretical analysis and experimental studies. *IEEE Trans. Fuzzy Syst.* 27 1543–1557. 10.1109/tfuzz.2018.2883022

[B76] Zhang Y. Ishibuchi H. Wang S. (2017). Deep takagi–sugeno–kang fuzzy classifier with shared linguistic fuzzy rules. *IEEE Trans. Fuzzy Syst.* 26:1. 10.1109/tcyb.2020.3016972 32946410

[B77] Zrenner C. Desideri D. Belardinelli P. Ziemann U. (2017). Real-time eeg-defined excitability states determine efficacy of tms-induced plasticity in human motor cortex. *Brain Stimul.* 11 374–389. 10.1016/j.brs.2017.11.016 29191438

